# *Wdfy3*-dependent autophagy impairment recapitulates presymptomatic neurodegenerative signatures in mice

**DOI:** 10.1038/s41598-026-43314-0

**Published:** 2026-04-04

**Authors:** Aldo Vorkapich, Arshi Mustafa, Amanda L. Flores-Torres, Konstantinos S. Zarbalis, Cecilia Giulivi

**Affiliations:** 1 Department of Molecular Biosciences, School of Veterinary Medicine, University of California, Davis, Davis, CA 95616 USA; 2 Department of Pathology and Laboratory Medicine, University of California, Davis, Sacramento, CA 95817 USA; 3 Institute for Pediatric Regenerative Medicine, Shriners Hospitals for Children, Northern California, 2425 Stockton Boulevard, Sacramento, CA 95817 USA; 4 Medical Investigations of Neurodevelopmental Disorders (M.I.N.D.) Institute, University of California, Davis, Sacramento, CA 95817 USA

**Keywords:** Parkinson’s disease, Mouse model, Autophagy, Mitochondrial dysfunction, Proteomics, Neurodegeneration, Biomarkers, Diseases, Neurology, Neuroscience

## Abstract

WDFY3/ALFY is an adaptor protein involved in selective autophagy. Loss of Wdfy3 in mice causes severe deficits in neuronal health, and pathogenic mutations in *WDFY3* are associated with neurodevelopmental disorders in humans. As impaired autophagy is increasingly implicated in Parkinson’s disease (PD) and other neurodegenerative disorders, we investigated whether *Wdfy3* haploinsufficiency produces early molecular and cellular signatures of neurodegeneration in *Wdfy3*^*+/lacZ*^ mice, given that these diseases often exhibit presymptomatic alterations preceding overt clinical manifestations. Cortical tissue from 3-month-old presymptomatic mice showed significant proteomic overlap with both patient-derived PD cell lines and human brain proteomic datasets, particularly from the substantia nigra, underscoring the translational relevance of this model. Consistent with disease progression, immunofluorescence analyses of the cortex and substantia nigra from 14-month-old mice revealed significant dysregulation of multiple markers associated with neurodegeneration. Together, these findings demonstrate that impaired autophagy resulting from reduced Wdfy3 expression recapitulates key features of neurodegenerative disease at both early and later stages. By providing a platform to investigate presymptomatic pathogenic mechanisms, this model may inform the development and testing of future diagnostic and therapeutic strategies aimed at preserving neuronal health.

**Supplementary Information:**

The online version contains supplementary material available at 10.1038/s41598-026-43314-0.

## Introduction

Autophagy is a fundamental cellular process that maintains homeostasis by degrading and recycling damaged or misfolded proteins and clearing dysfunctional organelles. In addition to its housekeeping role, autophagy enables cells to adapt to metabolic stress and nutrient deprivation^1^. In the nervous system, autophagy is particularly critical for neuronal health, contributing to neural plasticity, synaptic health, and long-term cellular viability^2–4^. Disruption of autophagy can therefore predispose neurons to degeneration through the age-dependent accumulation of damaged organelles and toxic protein species^5,6^.

Autophagy is regulated by a complex network of proteins and signaling pathways that govern its initiation, progression, and execution^7–11^. Among these are proteins containing WD40-repeat (WDR) and Beige and Chediak-Higashi (BEACH) domains^12^, which are highly conserved across species and cell types^12,13^. Deficits or pathogenic mutations in BEACH-containing proteins^14^ are associated not only with developmental, emotional, cognitive, and epileptic disorders, but also with neurodegenerative diseases. Consistent with this, impaired autophagy has been implicated in pathological processes occurring both early in life, including neurodevelopmental^3,7^, and later during aging, contributing to the development and progression of neurodegenerative diseases^6,15^.

Neurodegenerative diseases such as Alzheimer’s disease (ALZ) and Parkinson’s disease (PD) are typically diagnosed only after the onset of overt clinical symptoms. By this stage, autophagic dysfunction may have been present for decades, allowing the progressive accumulation of protein aggregates and damaged organelles that characterize these conditions^16–18^. While impaired autophagy has been independently linked to disrupted neurodevelopment and neurodegeneration, its contribution to disease pathogenesis has not been longitudinally mapped across presymptomatic and clinical stages in human patients^2,6^. Identifying conserved molecular alterations across stages and models could therefore help validate animal and cell-based systems for studying early disease mechanisms.

In this context, we investigated the role of WDFY3, a BEACH and WDR-containing protein that functions as a central scaffold and adaptor linking autophagic machinery to degradation substrates in selective autophagy, including the clearance of protein aggregates and organelles via macroautophagy, mitophagy^4,14^, and glycophagy^4^. WDFY3 and its homologs are highly conserved across species^12,19^, and accumulating evidence suggests that WDFY3 plays an important role in neurodevelopment and neurodegeneration^19^. This is supported by findings from multiple animal models^4,12,14,19–23^ and from individuals harboring pathogenic mutations in *WDFY3*^22,24,25^. In mice, Wdfy3 is prominently expressed in the developing central nervous system and in the adult brain^14,19,23^. Homozygous loss of *Wdfy3* results in perinatal lethality and severe disruptions in prenatal neurogenesis, neuronal migration, and both long- and short-range neuronal connectivity^19,20,23^. In contrast, mice with heterozygous, partial *Wdfy3* loss (*Wdfy3*^*+/lacZ*^ ) survive to adulthood, but exhibit disrupted autophagy and overt neurodevelopmental anomalies^23^. These mice display altered expression of numerous proteins, including autophagy-related components involved in multiple degradation pathways^4,14^, impaired neurogenesis and neuroplasticity, altered forebrain morphology^23^, and increased age-dependent accumulation of damaged organelles and metabolic aggregates^4^. Notably, *Wdfy3* haploinsufficiency accelerates neurodegeneration in a Huntington’s disease (HD) mouse model^21^.

Based on these observations, we hypothesized that impaired autophagy resulting from *Wdfy3* haploinsufficiency would give rise to conserved molecular signatures of neurodegenerative disease in the brain cortices of young *Wdfy3*^+/lacZ^ mice, prior to the onset of overt neurological symptoms. To test this hypothesis, we complemented our in vivo analysis with a parallel investigation of fibroblasts derived from patients with PD and then validated it using publicly available PD proteomic datasets. Although fibroblasts cannot fully recapitulate neuron-specific aspects of autophagy (such as specialized cargo processing, synaptic demands, or neuron–glia interactions), they remain accessible and informative patient-derived models. Fibroblasts from patients with PD, HD, amyotrophic lateral sclerosis (ALS), and frontotemporal dementia consistently show alterations in autophagy, mitochondrial function, and redox balance, highlighting their value for identifying conserved cellular mechanisms relevant to neurodegeneration^26,27^. Nevertheless, the absence of neuronal architecture and signaling contexts necessitates cautious interpretation and complementary validation in neuronal or brain-derived systems.

Accordingly, we analyzed brain cortices from 3-month-old *Wdfy3*^+/lacZ^ mice, an age at which molecular alterations are expected to be subtle, allowing the identification of changes that precede neurodegenerative pathology. In parallel, we examined fibroblasts from PD patients carrying pathogenic mutations in autophagy-linked genes, including *LRRK2*, an autophagy-associated kinase, and *GBA*, a lysosomal enzyme that represents the most common genetic cause of familial PD^8–11,28,29^. Mutations in *GBA* are also linked to lysosomal storage disorders such as Gaucher’s disease^29^ and Lewy-body dementia^30^. Defective autophagy in PD is thought to contribute to the accumulation of alpha-synuclein (SNCA), loss of dopaminergic neurons, and progressive cognitive impairment. Together, fibroblasts and mouse brain cortices provide complementary systems for studying cellular stress and proteostasis in patient-derived material and vulnerable neural tissues, respectively. Identifying shared alterations—such as impaired autophagy, early metabolic imbalance, and subtle protein aggregation—may illuminate molecular events associated with presymptomatic stages of neurodegenerative diseases, including PD^2,31,32^.

Using untargeted proteomics as an unbiased approach for early-stage biomarker discovery^33^, we compared differentially expressed proteins in brain cortices from 3-month-old *Wdfy3*^+/lacZ^ mice with those identified in PD-derived fibroblasts and human PD brain datasets. This analysis revealed overlapping pathways associated with mitochondrial dysfunction, dysregulated autophagy, and neurofilament cytoskeletal alterations. Immunofluorescence analyses of the brain cortex and substantia nigra from 14-month-old *Wdfy3*^+/lacZ^ mice extended these findings, confirming significant dysregulation of neurofilament architecture, a hallmark of neurodegeneration.

Overall, the convergence of early cross-model, cross-cell-type molecular signatures with the later emergence of PD-derived fibroblasts and the subsequent development of neurodegenerative phenotypes supports the use of autophagy-impaired *Wdfy3*^+/lacZ^ mouse as a model for investigating early pathogenic mechanisms. These findings highlight autophagy-related pathways as promising targets for early detection, disease monitoring, and therapeutic intervention prior to irreversible neuronal loss^32,34^.

## Results

### Brain proteome validates neuronal and disease-relevant signatures

Untargeted proteomics was performed on the brain cortex of 3-month-old *Wdfy3*^+/lacZ^ and sex- and age-matched wild-type control mice (WT) (*n* = 7 male mice each). In both groups, 2444 unique proteins were identified (Supplementary Table 1), providing broad proteomic coverage and a robust foundation for downstream analyses. Initial quality control confirmed the dataset’s reliability, as proteins characteristic of the respective tissue of origin were well represented (cortex neurons; Supplementary Fig. 1; Supplementary Tables 2–4).

Beyond validating sample identity, enrichment analyses showed that many of the detected proteins were involved in cognitive and synaptic processes (Supplementary Fig. 2; Supplementary Tables 5–6), underscoring the dataset’s functional relevance for investigating neurological pathways. Moreover, numerous proteins were associated with human diseases, including neurodegenerative disorders (Supplementary Fig. 3; Supplementary Table 7). Together, these observations confirm that the proteomic profiles accurately reflected cortical tissue composition and capture biologically meaningful pathways linked to cognition and disease, providing a strong basis for identifying early molecular changes relevant to neurodegeneration.

### Enriched biological and disease pathways link *Wdfy3* haploinsufficiency to neurodegeneration

Differentially expressed proteins (DEPs) were defined using a significance of *P* ≤ 0.05 without applying a log₂(fold-change) cutoff. This approach was chosen to enable detection of subtle, early alterations in protein abundance that may be biologically meaningful in presymptomatic models of neurodegeneration, where large fold changes are uncommon. Minor perturbations in key pathways—such as autophagy, proteostasis, or metabolism—may exert cumulative or network-level effects, and applying an arbitrary fold-change threshold could exclude proteins critical for understanding early disease mechanisms. By focusing on statistical significance, we aimed to capture nuanced molecular events that may be conserved across species and experimental models.

**Fig. 1:**
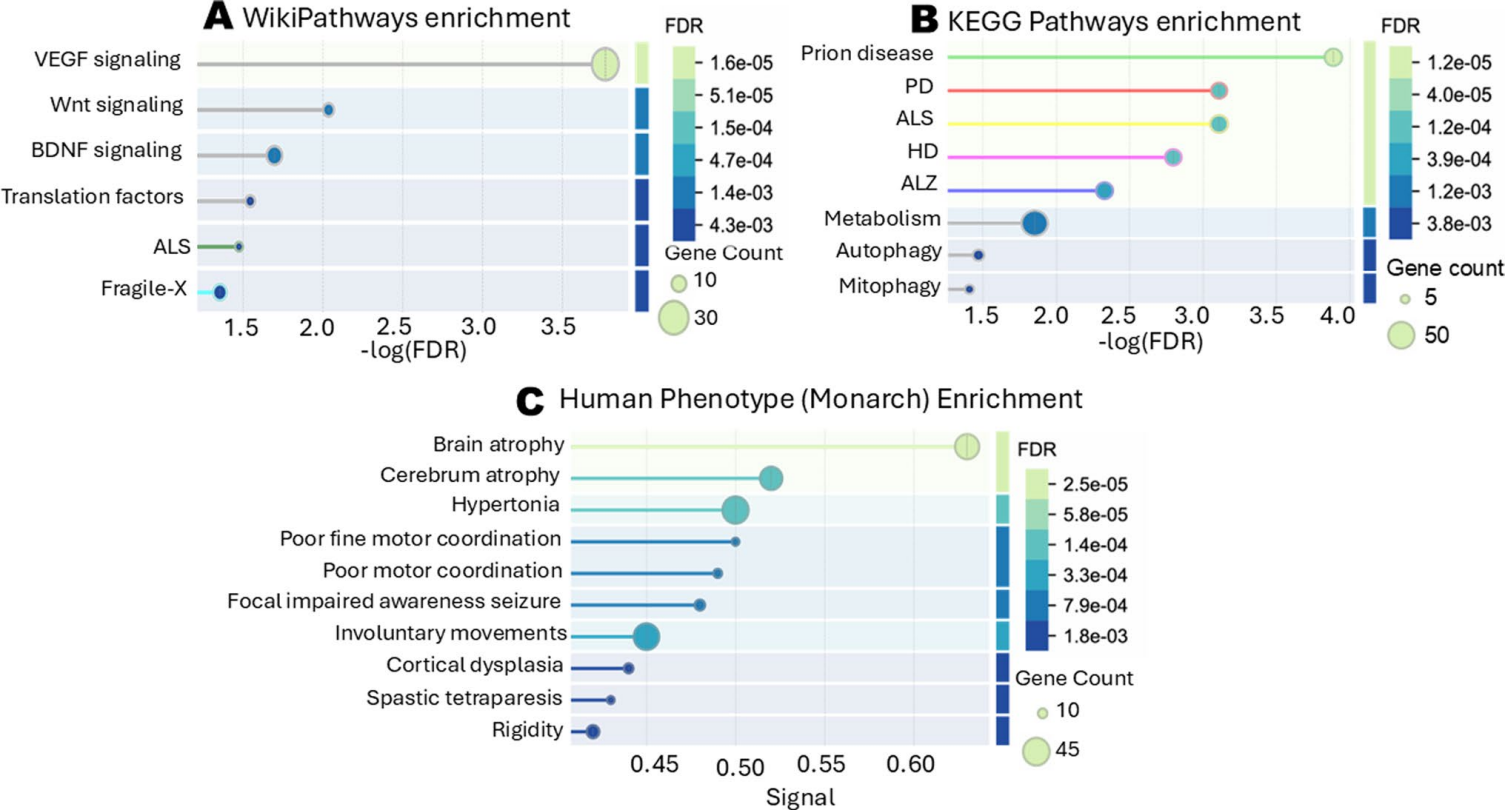
Differentially expressed proteins highlight autophagy, metabolism, and neurodegenerative pathways in *Wdfy3*^+/lacZ^ mice. STRING functional enrichment tool analysis of 414 DEPs between *Wdfy3*^+/lacZ^ mice and controls (*p* ≤ 0.05, Student’s *t*-test; Supplementary Table 8). The analysis encompassed biological pathways from 3 databases: (**A**) WikiPathways, (**B**) KEGG, and (**C**) Human Phenotype (Monarch). The functional enrichment visualization settings were configured as follows: group similarity terms threshold ≥ 0.4; number of terms displayed: 25; sorting criterion: –log FDR; minimum signal threshold ≥ 0.01; minimum count in network: 2; minimum strength threshold ≥ 0.01; FDR cut-off: ≤ 0.05 (Supplementary Tables 9–11 for a complete list of associations from each database).

Using these criteria, 414 DEPs were identified between *Wdfy3*^+/lacZ^ and WT cortices (Supplementary Tables 8–11; Fig. 1A). KEGG pathway analysis^35,36^, highlighted neurodegenerative proteopathies as the most significantly enriched categories, with prion disease and PD ranking highest, followed by ALS, HD, and ALZ. Additional enriched pathways included cellular metabolism, bioenergetics, and autophagy-related processes (e.g., Atg16l1, Ist1, Vps13c), including mitophagy (e.g., Bcl2l13; Fig. 1B; Supplementary Table 10). These results indicate that the observed protein changes resulting from *Wdfy3* haploinsufficiency were strongly linked to early neurodegenerative mechanisms.

Enrichment analysis using the Human Phenotype (Monarch) database further revealed associations with morphological and motor features commonly observed in neurodegenerative conditions, including brain atrophy, impaired fine motor coordination, rigidity, cortical dysplasia, and spastic tetraparesis (Fig. 1C). Collectively, these findings reinforce a connection *Wdfy3* haploinsufficiency, disrupted autophagy and mitophagy, and molecular signatures of neurodegenerative disease.

### Shared proteomic signatures in *Wdfy3*^+/lacZ^ and PD fibroblasts reveal common mechanisms in neurodegeneration

Given the strong association between the *Wdfy3*^+/lacZ^ mouse proteome and neurodegeneration-related pathways, particularly PD, we next assessed whether similar molecular signatures were present in human biospecimens from patients with PD-carrying autophagy-related mutations. Untargeted proteomics was performed on fibroblasts from PD patients harboring *GBA* and/or *LRRK2* mutations and age- and sex-matched controls (4 lines per group; Table 1). A total of 2173 unique proteins were identified, of which 1,766 were differentially expressed across diagnostic groups (Supplementary Table 12).

**Table 1:** Characteristics of PD cell lines used in this study*.

ID	Demographics		Genotype		Phenotypic characteristics					
	Sex	Age in years (at diagnosis)	Mutation	Chromosome location	Bradykinesia	Gait difficulties	Postural instability	Anti-PD therapy	Resting tremor	Rigidity
ND-34,980	M	58 (54)	LRRK2-G2019S; GBA-N370S	12q12, 1q21	Y	na	na	R	Y	Y
ND-29,802	M	52 (40)	LRRK2-G2019S	12q12	Y	Y	na	R	Y	Y
ND-34,263	M	65 (40)	GBA-N370S	1q21	Y	Y	Y	R	Y	Y
ND-41,015	M	52 (42)	GBA-E326K homozygous	1q21	Y	na	na	na	na	na

Background information on fibroblast cell lines derived from human PD patients, including patient phenotypes, clinical information, and the genetics of the cell line.

*M, male; Y = yes; na, not available; R = tried and responsive.

**Fig. 2:**
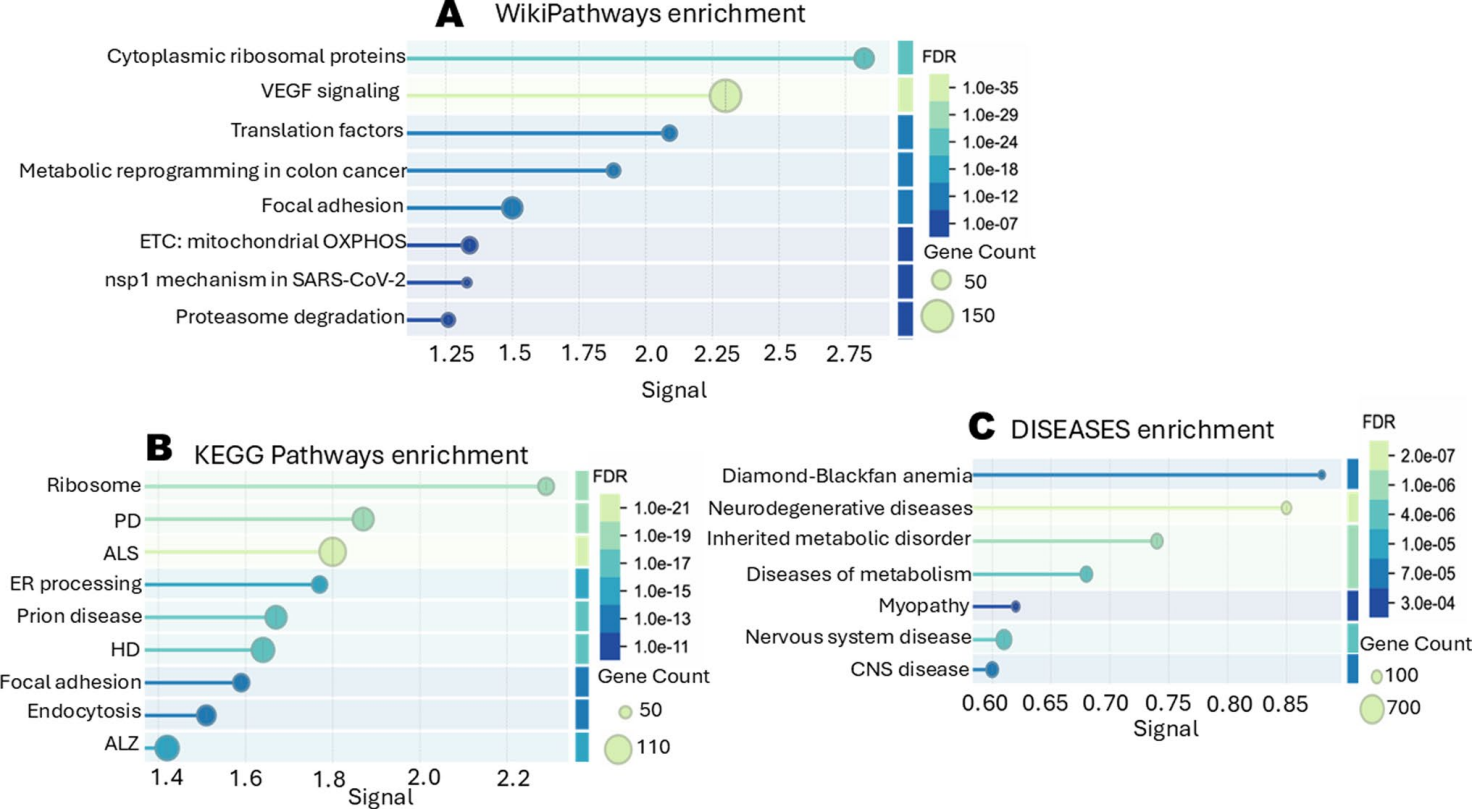
Differentially expressed proteins highlight autophagy, metabolism, and neurodegenerative pathways in human PD fibroblasts. STRING functional enrichment tool analysis of 1766 DEPs between PD and control human fibroblasts (demographics in Table 1; Supplementary Table 12 for *t*-tests). The analysis encompassed biological pathways from three databases: (**A**) WikiPathways, (**B**) KEGG, and (**C**) DISEASES (JensenLab) (Supplementary Table 13 for a complete list of associations from each database).

Pathway enrichment analysis revealed consistent patterns across databases. WikiPathways highlighted protein translation, post-translational processing, mitochondrial function, metabolism, and proteasomal degradation (Fig. 2A). KEGG analysis^35,36^, further confirmed enrichment of neurodegenerative disease pathways—including PD, ALS, prion disease, HD, and ALZ—alongside carbon metabolism and autophagy-related pathways (Fig. 2B). The DISEASES database similarly identified neurological, metabolic, and neurodegenerative disease associations (Fig. 2C; Supplementary Table 13).

**Fig. 3:**
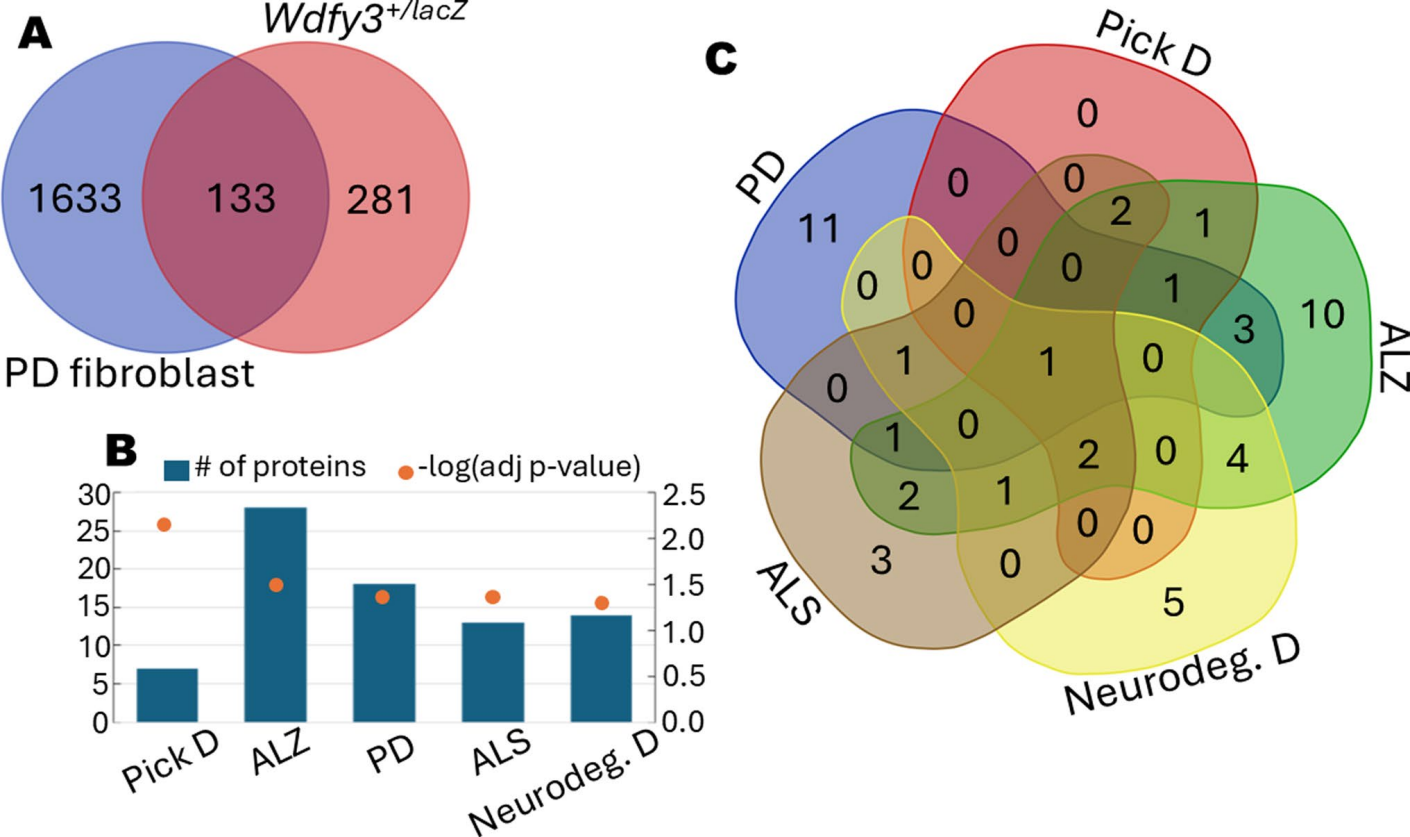
Conserved neurodegenerative protein signatures in PD and *Wdfy3*^+/lacZ^ models. (**A**) Venn diagram showing the 133 overlapping DEPs shared by PD vs. control human cells, and *Wdfy3*^+/lacZ^ vs. WT mice (Supplementary Table 14 lists specific DEPs). (**B**) Bar diagram depicting the results of DisGeNET (under EnrichR) analysis utilized to identify enriched diseases associated with the 133 proteins at the intersection between PD and *Wdfy3*^+/lacZ^ (only shown those filtered by adj *p*-value ≤ 0.05 and by neurodegeneration). The number of associated proteins is indicated on the left y-axis. In contrast, the negative log of respective *p*-values is shown as orange dots and indicated on the right y-axis (Supplementary Table 15 for a complete list of associations). (**C**) Venn diagram of the disease-associated proteins from diagram B, with 19 exhibiting overlapping associations with at least two neurodegenerative diseases.

Direct comparison of DEPs from mouse cortex and PD fibroblasts revealed 133 shared proteins (Fig. 3A; Supplementary Table 14). Disease enrichment analysis using DisGeNET (via EnrichR) linked these proteins to Pick disease, ALZ, PD, and ALS (Fig. 3B; Supplementary Table 15), with 19 contributing to at least two disease associations (Supplementary Table 16). Notably, three of these proteins (ALB, PRDX2, PRDX6) were also differentially expressed in the temporal lobes of patients with tauopathies^37^.

**Fig. 4:**
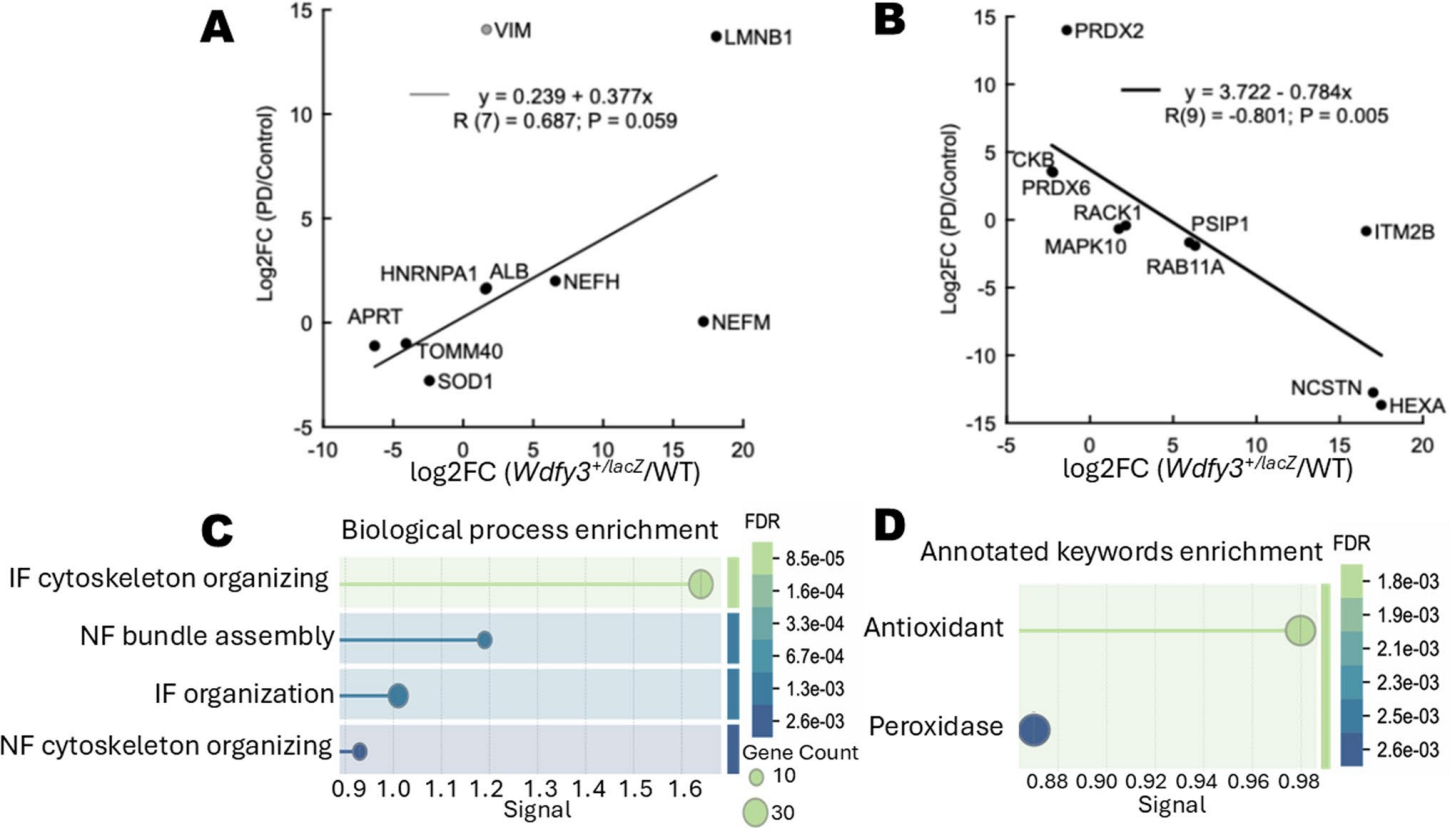
Shared neurodegeneration-associated proteins show positive and inverse correlations with distinct pathway enrichments. Of the 19 proteins described in Fig. 6C, nine exhibited positively correlated enrichments between mice (*Wdfy3*^+/lacZ^ vs. WT) and human fibroblasts (PD vs. control) (**A**), and 10 exhibited inversely correlated enrichments between species (**B**). The fold change for each protein was plotted by model, and linear regression was performed, showing significance for both subsets (Huber M-estimation *p* = 0.0019 and *p* < 0.0001, respectively). STRING analysis (GO: Biological Process) revealed enriched pathways associated with each subset. The two subsets were analyzed independently with functional enrichment visualization settings configured as follows: group similarity terms threshold ≥ 0.8; displayed number of terms: 3; sorting criterion: signal; minimum signal threshold ≥ 0.01; minimum count in the network: 2; minimum strength threshold ≥ 0.01; FDR cut-off: ≤ 0.05. The nine positively correlated proteins yielded enrichment for IF and neurofilament pathways (**C**), and the 10 inversely correlated proteins were enriched for antioxidant pathways (**D**).

Among the 19 disease-associated proteins, nine exhibited concordant differential expression in both models (Fig. 4A), whereas ten were inversely correlated (Fig. 4B). STRING analysis showed that the positively correlated proteins were enriched for intermediate filament cytoskeleton organization and neurofilament (NF) bundle assembly (LMNB1, NEFH, NfM, VIM), along with reduced expression of the antioxidant enzyme SOD1 (Fig. 4C). These findings supported a role for NF dysregulation in early neurodegeneration and its potential utility as a biomarker of white matter damage^38,39^. In contrast, inversely correlated proteins were enriched in antioxidant defense pathways (Fig. 4D), with PRDX2 and PRDX6 increased in PD fibroblasts but reduced in *Wdfy3*^+/lacZ^ mouse cortex, suggesting divergent oxidative stress responses across models.

## Overlap of *Wdfy3*^+/lacZ^ cortical proteome with public PD datasets

To further contextualize our findings, we compared DEPs from *Wdfy3*^+/lacZ^ mouse cortex with publicly available human PD brain and cerebrospinal fluid (CSF) proteomic datasets, including substantia nigra (PXD037684), prefrontal cortex (PXD030142), frontal cortex (PXD007160), and CSF (PPI_177) (Supplementary Tables 17–19). Statistically significant overlap was observed with two datasets: 65 proteins overlapping with substantia nigra and four proteins with prefrontal cortex datasets (Fisher’s exact test, *P* = 8 × 10^−12^ and *P* = 0.042, respectively; Supplementary Table 20).

Given the more substantial overlap with the substantia nigra dataset, the 65 shared proteins were subjected to pathway enrichment analysis using DAVID (Supplementary Table 21). The top enriched pathways included membrane trafficking, vesicle transport, trans-Golgi network vesicle budding, lysosome biogenesis, prion disease, HSP90-mediated protein folding, macro- and selective autophagy, cellular response to stress, regulation of necrosis, and translation (Supplementary Table 22). These results indicated that early proteomic changes in *Wdfy3*^+/lacZ^ cortex recapitulated key molecular pathways implicated in human PD, particularly within vulnerable substantia nigra neurons, reinforcing the translational relevance of this mouse model.

## Increased expression of neurodegeneration-associated markers in the cortex of older *Wdfy3*^+/lacZ^ mice

Although α-synuclein (SNCA) aggregation is central to PD pathology, increasing evidence implicates cytoskeletal proteins, including neurofilaments (NFs), in disease progression^40^. To determine whether early proteomic signatures of intermediate filament and neurofilament dysregulation correspond to histologically detectable changes, we examined the brains of 14-month-old *Wdfy3*^+/lacZ^ mice. We focused on SNCA as well as α-internexin (INA) and medium neurofilament (NfM), type IV neuronal intermediate filaments, that predominate at early and late stages of normal neuronal maturation, respectively^41^.

**Fig. 5:**
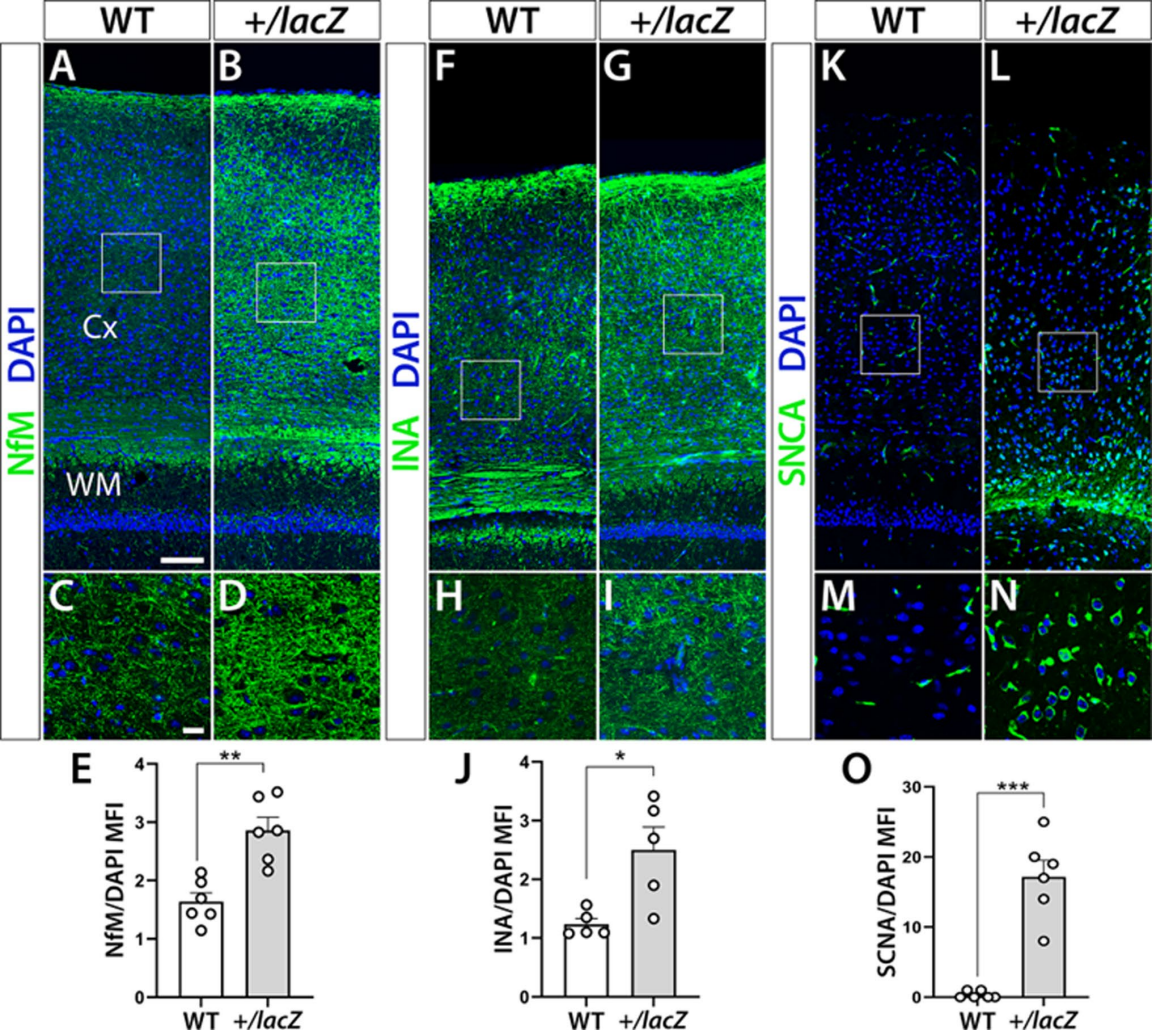
Genotype-dependent alterations in neuronal protein markers in the cortex of 14-month-old Wdfy3^+/lacZ^ mice. Coronal brain sections of cortex from 14-month-old WT and *Wdfy3*^*+/lacZ*^ mice were subjected to immunofluorescence analysis for NfM (**A–E**), INA (**F–J**), and SNCA (**K–O**). DAPI served as a nuclear counterstain to scale protein expression by cell count. White squares in the low magnification images (upper panels) indicate the positions of the corresponding high magnification images shown below. (**E**, **J**, **O**) Bar diagrams depicting the results of quantifications and statistical analyses of NfM, INA, and SNCA, respectively. *p < 0.05, **p < 0.01, ***p < 0.001 indicate statistically significant differences between genotypes. WT: wild-type controls, *+/lacZ*: *Wdfy3*^*+/lacZ*^, NfM: neurofilament medium chain, INA: alpha-internexin, SNCA: alpha-synuclein, Cx: cerebral cortex, WM: white matter. Scale bar in (**A**) is 100 μm and in (**C**) 20 μm.

Immunofluorescence analysis revealed significantly increased expression of all three markers in the cerebral cortex from 14-molth-old *Wdfy3*^*+/lacZ*^ mice. NfM expression was significantly higher in *Wdfy3*^*+/lacZ*^ mice (NfM/DAPI mean fluorescence intensity (MFI) 2.86 ± 0.22) compared with age- and sex-matched WT mice [MFI 1.64 ± 0.15; *t* (10) = 4.54, *p* < 0.01; Fig. 5A–E]. Similarly, INA cortical expression was higher in *Wdfy3*^*+/lacZ*^ mice (INA/DAPI MFI 2.50 ± 0.40) compared with WT (MFI 1.24 ± 0.10; *p* < 0.05; Fig. 5F–J]. SNCA expression was particularly increased in *Wdfy3*^*+/lacZ*^ mice (SNCA/DAPI MFI 18.6 ± 1.4) compared with WT (MFI 0.33 ± 0.21; Mann-Whitney U = 0.000, *p* = 0.01; Fig. 5K–O].

These histological findings at 14 months of age provided a tissue-level correlate to the early proteomic enrichment of NF bundle assembly and intermediate filament organization observed in 3-month-old *Wdfy3*^+/lacZ^ mice and PD-derived fibroblasts.

## Altered tyrosine hydroxylase and neurofilament expression in substantia nigra of older *Wdfy3*^+/lacZ^ mice

A defining pathological feature of PD is degeneration of dopaminergic neurons in the substantia nigra pars compacta (SNc), which are characterized by high tyrosine hydroxylase (TH) expression. Accumulation of misfolded proteins damages these selectively vulnerable neurons, leading to neuronal loss and reduced TH expression. To assess whether *Wdfy3* haploinsufficiency affects these markers, we examined TH, NfM, INA, and SNCA expression in the substantia nigra of the same 14-moth-old mice used for cortical analyses.

**Fig. 6:**
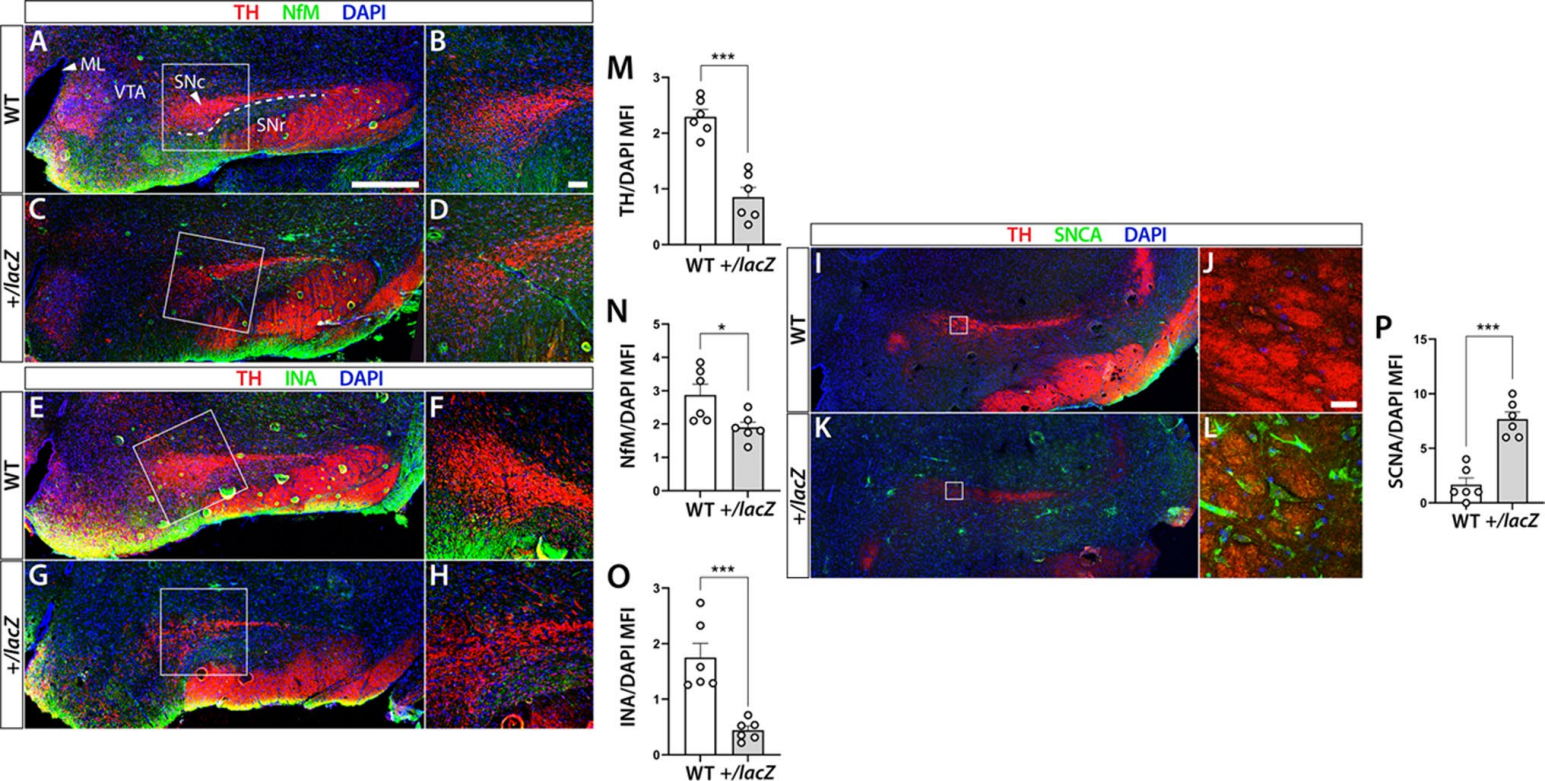
Genotype-dependent alterations in neuronal protein markers in the substantia nigra of 14-month-old *Wdfy3*^+/lacZ^ mice. Coronal brain sections of substantia nigra (SN) of 14-month-old WT and *Wdfy3*^*+/lacZ*^ mice were subject to immunofluorescence analysis for TH (**A–M**), NfM (**A–D**, **N**), INA (**E–H**, **O**), and SNCA (**I–L**, **P**). DAPI served as a nuclear counterstain to scale protein expression by cell count. White squares in the low-magnification images indicate the positions of the corresponding high-magnification images shown to the right. Bar diagrams depicting the results of quantifications and statistical analyses of TH, NfM, INA, and SNCA, respectively (**M–P**). *p < 0.05, ***p < 0.001 indicate statistically significant differences between genotypes. WT: wild-type controls, *+/lacZ*: *Wdfy3*^*+/lacZ*^, TH: tyrosine hydroxylase, NfM: neurofilament medium chain, INA: alpha-internexin, SNCA: alpha-synuclein, ML: midline, SNc: substantia nigra pars compacta, SNr: substantia nigra pars reticulata, VTA: ventral tegmental area. Scale bar in (**A**) is 500 μm, in (**B**) 100 μm, and in (**J**) 20 μm.

In the SNc, TH expression was significantly reduced in *Wdfy3*^*+/lacZ*^ mice (0.85 ± 0.17) compared with age- and sex-matched WT mice [2.29 ± 0.13; *t* (10) = 6.63, *p* < 0.001; Fig. 6A–L, M]. NfM expression was also decreased in *Wdfy3*^*+/lacZ*^ mice (1.81 ± 0.16) relative to WT [2.67 ± 0.25; *t* (10) = 2.9, *p* < 0.05; Fig. 6A–D, N]. INA expression similarly declined in *Wdfy3*^*+/lacZ*^ (INA/DAPI MFI 0.44 ± 0.07) compared with WT [MFI 1.75 ± 0.26; *t* (10) = 4.9, *p* < 0.001; Fig. 6E–H, O]. In contrast, and consistent with the cortical findings, SNCA expression was markedly increased in the SNc of *Wdfy3*^+/lacZ^ mice (SNCA/DAPI MFI 7.67 ± 0.67) relative to controls [1.67 ± 0.61; *t* (10) = 6.617, *p* < 0.001; Fig. 6I–L, P].

Together, these results demonstrated region-specific and marker-specific dysregulation in older *Wdfy3*^+/lacZ^ mice, further supporting the relevance of this model for studying autophagy-related mechanisms underlying PD and other neurodegenerative diseases.

## Discussion

We identified proteomic alterations in presymptomatic 3-month-old *Wdfy3*^+/lacZ^ mice—chronologically analogous to 20–30-year-old humans^42^—that reflect pathways implicated in neurodegeneration. Remarkably, these early changes overlapped significantly with those observed in samples from older PD patients, despite the human donors being symptomatic and decades older. Immunofluorescence analysis in the cortices of 14-month-old mice *Wdfy3*^+/lacZ^ mice — analogous to late middle-age (fifth decade of life)^42^—revealed increased expression of disease-associated proteins, providing a histological correlate to the early proteomic enrichment in NF bundle assembly and intermediate filament pathways. Further analysis of the substantia nigra uncovered dysregulated expression of markers with particular relevance to PD^43^. Together, these findings support the idea that autophagy-associated molecular disruptions can emerge long before clinical manifestation^17^, underscoring the importance of early intervention and highlighting the *Wdfy3*^+/lacZ^ model as a platform for studying preclinical neurodegenerative mechanisms^31,44^.

Proteomic enrichment of autophagy pathways was accompanied by altered expression of specific autophagy-linked proteins in *Wdfy3*^+/lacZ^ mice, including Atg16l1, Bcl2l13, Ist1, and Vps13c (Supplementary Tables 8, 10). Atg16l1, a core component of autophagosome formation, was underrepresented in *Wdfy3*^+/lacZ^ mice, consistent with previous findings^4^. Bcl2l13, a key promoter of mitophagy, also showed reduced expression, suggesting impaired mitochondrial quality control^45^. Conversely, Ist1—a facilitator of autophagosome–lysosome fusion—was upregulated, potentially indicating compensatory responses involving endosomal recycling in the setting of impaired autophagy^46^. Reduced Vps13c, which has been implicated in lysosomal homeostasis and early-onset PD, likely further compromises lysosomal integrity^47^. Collectively, these changes indicate that *Wdfy3* haploinsufficiency disrupts multiple nodes within the autophagy network, linking defective cargo clearance to secondary cellular stress pathways.

Building on this, proteomic and histological analyses pointed to overlapping dysregulation of NF proteins and antioxidant enzymes—key contributors to early neurofibrillary pathology—in *Wdfy3*^+/lacZ^ mice and PD fibroblasts. Elevated intermediate filament proteins (LMNB1, NfM, VIM, and INA) together with reduced SOD1 expression suggest concurrent cytoskeletal remodeling and weakened antioxidant buffering capacity. LMNB1 dysregulation has been associated with nuclear dysfunction in ALZ^48,49^ and LRRK2-PD^48^, while NEFH and NfM support axonal stability and contribute to mitochondrial scaffolding^50^, and they colocalize with tau within neurofibrillary tangles across several neurodegenerative conditions^51–54^. Importantly, NF proteins are released into CSF and blood during axonal degeneration, serving as biomarkers of presymptomatic neurodegenerative progression^55,56^. Similarly, VIM and INA contribute to early cytoskeletal dynamics and mitochondrial trafficking^57,58^. INA is implicated in inclusion formation in neurodegenerative disorders, including neuronal intermediate filament inclusion disease (NIFID)^59^, and is partly replaced by NfM during normal neuronal maturation^41^. Altered expression of both proteins has been linked to neuronal injury^60^ and neurodegenerative disorders^59,61^. Together, these findings suggest that impaired autophagy in *Wdfy3*^+/lacZ^ mice destabilizes the cytoskeletal organization, thereby coupling defective protein clearance to axonal and mitochondrial dysfunction. This convergence of autophagy and cytoskeletal pathways across species and models underscores conserved mechanisms of neurodegenerative vulnerability.

Consistent with this link, the antioxidant enzymes PRDX2, PRDX6, and SOD1 showed model-specific alterations, further linking autophagy deficiency to redox imbalance. Reduced SOD1 in both models may reflect impaired antioxidant capacity associated with NF dysregulation, consistent with evidence that deletion of *NEFL* reduces motor neuron vulnerability and slows disease progression in *Sod1* mutant mice^62^. This interaction underscores the interdependence between oxidative stress and cytoskeletal integrity in neurodegeneration^63^. Meanwhile, decreased PRDX2 and PRDX6 in *Wdfy3*^+/lacZ^ mice may exacerbate ROS accumulation and impair mitophagy^4,14,64^, whereas their elevation in PD fibroblasts may reflect tissue-specific compensatory responses^65–67^.

To assess the translational relevance of the *Wdfy3*^+/lacZ^ model, we compared cortical DEPs with publicly available human PD proteomes. The strongest overlap was observed with substantia nigra datasets, highlighting conservation of autophagy, vesicle trafficking, proteostasis, and stress-response pathways across species and brain regions. Thus, *Wdfy3* haploinsufficiency appears to induce early molecular changes resembling those naturally enriched in substantia nigra from PD patients. In this sense, dysregulation of autophagy, oxidative stress responses, and NF organization in *Wdfy3*^+/lacZ^ cortex may “prime” cortical tissue with substantia nigra-like molecular signatures. By contrast, human cortical datasets may dilute these disease-relevant signals due to cellular heterogeneity, potentially contributing to weaker overlap. These findings underscore that Wdfy3 deficiency shifts cortical proteomic profiles toward pathways characteristic of vulnerable nigral neurons, highlighting conserved early mechanisms of neurodegeneration.

To explore the downstream effects of early proteomic changes, we analyzed protein expression at 14 months and observed region-specific changes. Cortex featured increased levels of NfM, INA, and SNCA. In contrast, SNc shows reduced expression of TH, INA, and NfM, alongside increased SNCA, consistent with a PD-like pattern affecting nigral dopaminergic neurons. Post-mortem studies of substantia nigra from PD patients show pronounced loss of non-phosphorylated NF immunoreactivity in surviving neurons, indicating disruption of NF integrity during neurodegeneration^43^. Proteomic analyses further reveal decreased abundance of NfM chains in PD substantia nigra tissue compared with controls, supporting a role for NF pathology in nigrostriatal degeneration^43,68,69^. Evidence also indicates that NfM physically associates with INA and other NF proteins, contributing to NF assembly and axonal transport^70^. Importantly, NF proteins, including NfM and INA, can aggregate with SNCA in Lewy bodies, suggesting functional interplay between intermediate filament aggregation and SNCA pathology in PD^71^. Intermediate filament aggregation is also a feature of other neurodegenerative disorders, implying that shared mechanisms of cytoskeletal disruption may underlie neuronal vulnerability and SNCA-associated toxicity^59^.

Our study highlights Wdfy3 as a pivotal regulator of autophagy and mitophagy, essential for maintaining neuronal energy balance and structural integrity^4,14,20,22,23^. Reduced Wdfy3 disrupts mitochondrial turnover^4^ and accelerates early cellular pathology^20,21,23^, bridging neurodevelopmental processes with preclinical neurodegenerative phenotypes, and contributing to the later emergence of region-specific neurodegenerative disease markers.

**Fig. 7:**
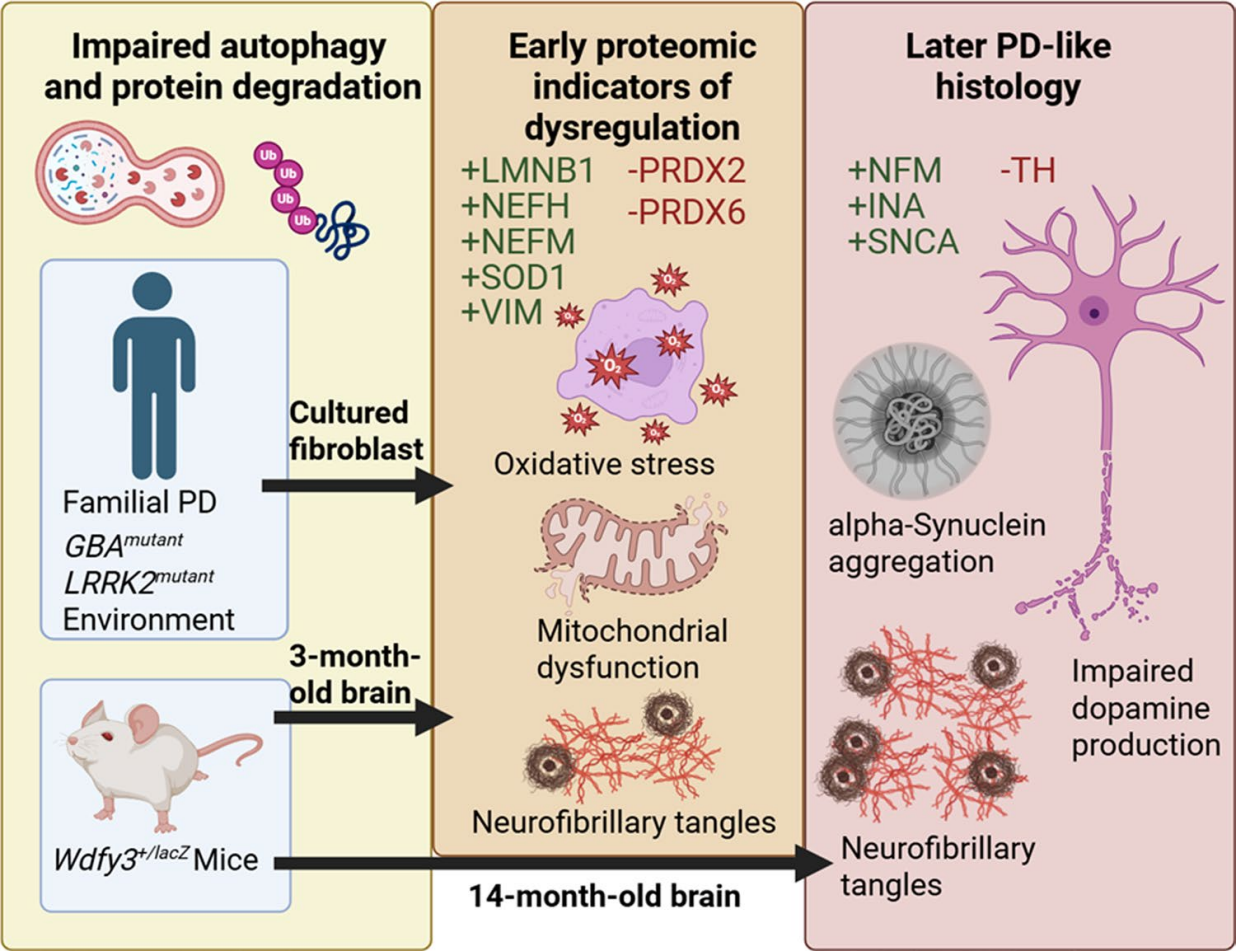
Impaired autophagy and downstream effects in mouse and human fibroblast models. Haploinsufficiency of Wdfy3 in *Wdfy3*^*+/lacZ*^ mice impairs autophagy, while *GBA* and *LRRK2* mutations cause similar defects in human PD (Yellow Panel). In both 3-month-old *Wdfy3*^*+/lacZ*^ mice and PD-derived fibroblasts, impaired autophagy triggers cascading proteomic alterations (e.g. Fig. 4) associated with oxidative stress, mitochondrial dysfunction, neurofilament dysregulation, and the early formation of tangles (Orange Panel; “+” proteins are positively correlated between models and “–“ the opposite). Impaired autophagy also contributed to NfM, INA, and SNCA aggregation in the cortex of 14-month-old *Wdfy3*^*+/lacZ*^ mice, and elevated SNCA with reduced TH in SNc, linking impaired autophagy to downstream neuronal dysfunction and disease-relevant phenotypes (Figs. 1, 2, 3, 4, 5 and 6; Red Panel) “+” proteins are elevated in *Wdfy3*^*+/lacZ*^ and “–“ reduced). These findings highlight conserved mechanisms across species and model systems. NfM: neurofilament medium chain, INA: alpha-internexin, SNCA: alpha-synuclein, TH: tyrosine hydroxylase, SNc: substantia nigra pars compacta. Graphics created in https://BioRender.com.

In summary, autophagy-impaired young *Wdfy3*^+/lacZ^ mice recapitulate early molecular and structural hallmarks of human neurodegeneration, providing a powerful platform for identifying pathogenic mechanisms and therapeutic targets before overt clinical manifestation. Older *Wdfy3*^+/lacZ^ mice show brain-region-specific alterations in protein expression, illustrating how impaired autophagy can drive downstream neurodegenerative processes. Given that defective autophagy contributes to PD and other neurodegenerative disorders^2,6,15^—further shaped by gene–environment interactions^72–74^—targeting autophagy-related pathways holds promise for early detection, intervention, and preservation of neuronal resilience^31,32,44^ (See graphical abstract: Fig. 7).

## Materials and methods

### Animal breeding and husbandry

Animal work was conducted in strict alignment with the ARRIVE (Animal Research: Reporting of In Vivo Experiments) guidelines^75^ to ensure transparency, reproducibility, and ethical integrity. We clearly defined all experimental procedures, including animal species, strain, sex, and age, and provided detailed descriptions of the housing and environmental conditions. Sample sizes were determined through statistical power analysis to minimize animal use while ensuring data reliability. All experimental interventions, including handling and observation, were designed to minimize stress and discomfort, according to ethical guidelines approved by the institutional animal care committee. Outcomes were reported comprehensively, capturing both primary and secondary findings to support thorough interpretation and future reproducibility of our research.

*Wdfy3*^*+/lacZ*^ *(Wdfy3*^*tm1a(KOMP)Mbp*^) mice were generated and genotyped as previously described^23^ and maintained on a C57BL/6NJ background as a mixed WT/heterozygous mutant colony in facilities approved by the Association for Assessment and Accreditation of Laboratory Animal Care International (AAALAC). All mice were genotyped for *Nnt* as described before^14^. In brief, *Wdfy3*^*+/lacZ*^ mouse generation using a knock-in “knockout-first” lacZ reporter construct inserted between exons 7 and 8 of the *Wdfy3* gene was previously described^23^. Mice were housed as a mixed WT and *Wdfy3*^*+/lacZ*^ colony in Plexiglas cages (55 × 33 × 19 cm) and maintained in facilities approved by the AAALAC under standard laboratory conditions (21 ± 2 °C; 55 ± 5% humidity) on a 12-h light/dark cycle, with ad libitum access to both water and standard rodent chow. The University of California approved animal handling protocols, Davis Institutional Animal Care and Use Committee, overseen by the AAALAC accreditation program, and are in compliance with ARRIVE^75^ and NIH guidelines^76^.

### Subject samples

All fibroblasts were obtained from the Coriell Institute for Medical Research. Fibroblast cell lines were derived from 4 male patients with PD (Table 1). These cell lines were matched for age and sex with another four cell lines from non-PD individuals aged 59 to 67 years. The cells were grown to 75–80% confluency to evaluate their proteome. The cell passage for all measurements was kept to a minimum. Cells were grown according to Coriell’s instructions (DMEM with 2 mM Gln) with 15% fetal bovine serum (Invitrogen), 1× penicillin/streptomycin/glutamine (Invitrogen), 1% non-essential amino acids (Invitrogen), and 1:250 fungizone (J R Scientific, Woodland, CA, USA).

### Untargeted proteomics

Proteins from cortices from age- and sex-matched (all males) WT and *Wdfy3*^*+/lacZ*^ mice (*n* = 7/group) at 3 months of age, as well as from cultured human dermal fibroblasts from control and PD patients (Table 1). Sample separation and concentration were performed as previously described^14^. Proteomic profiling of cultured cells included three independent biological replicates, each analyzed in duplicate. Proteomic analysis of mouse cortices consisted of a single biological experiment with duplicate measurements. Briefly, after homogenization in PBS, large cell debris and nuclei were pelleted by centrifugation at 600 x g for 5 min. The supernatants were concentrated by acetone precipitation and submitted to untargeted proteomics performed at the UCD Mass Spectrometry Core facility as described before^14^. Proteins were identified using a false discovery rate (FDR) of 1% at both the peptide-spectrum match and protein levels, with a minimum of two unique peptides required for protein identification.

### Enrichment analysis

Where indicated, the complete ranked proteomic dataset was uploaded to STRING (v12.0) for network visualization and enrichment analysis. In this mode, STRING interprets protein rank or abundance data to assign weights to nodes within the interaction network. Therefore, inclusion of all detected proteins—rather than only statistically significant ones—is essential to preserve network connectivity and allow unbiased detection of interaction clusters and enrichment patterns. Limiting the input to significant hits can disrupt network structure, leading to false underrepresentation of biological pathways.

### Databases and resources utilized in this study

Role of BEACH-containing proteins in human diseases (Online Mendelian Inheritance in Man (OMIM), NHGRI-EBI Catalog of Human Genome-Wide Association Studies (GWAS), DatabasE of genomiC varIation and Phenotype in Humans using Ensemble Resources (DECIPHER), DisGeNET.

Analysis of human WDFY3 and homologous proteins across species: PhylomeDB

WDFY3 and MAPT gene expression in human and mouse brains: The Human Protein Atlas (HPA)

Cell and Tissue assignment of cortical brain proteome: EnrichR (PanglaoDB, Human Gene Atlas (HGA), Mouse Gene Atlas (MGA))

Cell component and process assignment of cortical brain proteome: SynGO

Disease/Pathway analysis of DEP: STRING, WikiPathways, REACTOME, DisGeNET, Human Phenotype (Monarch), DISEASES (JensenLab), KEGG Pathways^35,36^

Comparison with PD proteomic datasets: ProteomeXchange, PPMI (PPMI_177)

### Histology and immunofluorescent analysis

Mice (males, 14-month-old WT and *Wdfy3+/lacZ*) were transcardially perfused with phosphate-buffered saline (PBS) followed by 4% paraformaldehyde (PFA) in PBS. Perfusion took place under anesthesia delivered by isoflurane inhalation via a precision vaporizer. Anesthesia was induced using 3–5% isoflurane in oxygen in an induction chamber and maintained at 1–2% isoflurane in oxygen via a nose covering tube during surgery. Adequate anesthesia was confirmed by loss of the pedal withdrawal reflex, slow and regular breathing, and absence of response to a tail pinch. Subsequently, mice were decapitated, brains dissected, fixed overnight in PFA/PBS, washed in PBS, and then transferred to 15% and 30% sucrose in PBS. After brief incubation in OCT compound, tissues were frozen over dry ice and stored at -80 °C until sectioned. All immunofluorescence experiments were performed on slide-mounted sections. The sections were fixed for 8 min at 20–22 °C in 4% PFA/PBS, then washed in PBS three times for 5 min each. Heat-induced epitope retrieval was performed using a Biocare Medical Decloaking Chamber NxGen with the DIVA Decloaker solution (Biocare Medical, Pacheco, CA) diluted to the working concentration in PBS. Samples were washed in PBS and incubated in PBS containing 10% donkey serum and 0.1% Triton X-100 for one hour. Then, sections were incubated in 1% donkey serum + 0.1% Triton X-100 in PBS overnight at 4 °C with the following primary antibodies and dilutions: mouse α-NfM (Invitrogen, 13–0700, 1:200), rabbit α-INA (Abcam, EP676Y, 1:300), mouse α-SNCA (ThermoFisher, clone LB509, 1:200), and rabbit α-TH (Millipore, AB152, 1:400). Fluorophore-conjugated secondary antibodies were applied in same solution diluted 1:200 for 2 h at 20–22 °C. Sections were then washed, mounted using Fluoromount-G, and imaged at 10×, 20×, or 100× magnification using a Nikon A1 confocal microscope and associated NIS Elements software (Nikon Instruments Inc., Melville, NY). All images were acquired using identical acquisition parameters across experimental groups. Quantitative image analysis was performed on six brains per genotype and marker using ImageJ/Fiji, and all analyses were conducted blinded.

### Comparative analysis of *Wdfy3*^+/lacZ^ cortex proteome with human PD datasets

We identified two publicly available proteomic datasets from Parkinson’s disease (PD) human cerebrospinal fluid (CSF) and brain tissues: ProteomeXchange^77^ and PPMI (Parkinson’s Progression Markers Initiative). From ProteomeXchange, we selected three datasets: one from the substantia nigra, a primary brain region affected in PD, and two from the cortex, which corresponded to the murine tissues used in our proteomics analysis (Supplementary Table 17). Following the download, datasets were filtered to include only differentially expressed proteins (DEPs) as reported by the respective authors. PPMI was excluded from this step, as it lacked a linked publication. We then quantified the overlap between our protein list and each public dataset utilizing a Venn diagram^78^ (Supplementary Tables 18–19). To assess whether the observed overlaps were statistically significant, Fisher’s exact test was performed, and the odds ratios were calculated (Supplementary Table 20). Finally, pathway enrichment analysis of the overlapping proteins from PXD37684 was conducted using KEGG^79^ and REACTOME pathway databases via DAVID (Database for Annotation, Visualization, and Integrated Discovery^80^, to identify shared biological processes and pathways.

### Statistical analysis

Statistical significance was set at *p* ≤ 0.05, and data analysis was performed using JMP Pro v17 to ensure consistency and reproducibility of the results. Histological data were first tested for normality using the Shapiro-Wilk test. To examine genotype differences within each brain region, a *t*-test was used for normally distributed data and a Mann–Whitney U test for non-normally distributed data. Data are presented as mean ± SEM.

### Ethics and editorial policies statements

*Ethics declaration*. The University of California, Davis Institutional Animal Care and Use Committee (IACUC) for the Protection of Animal Welfare (#21780) approved all experimental procedures and the ethics of this study. The study was conducted in accordance with IUCAC requirements and the ARRIVE guidelines^75^.

## Supplementary Information

Below is the link to the electronic supplementary material.

Supplementary Material 1

Supplementary Material 2

## Data Availability

The study did not generate new unique reagents. All data generated or analyzed during this study are included in this published article and under Supplemental Information .
