# Supplementary material for: Wdfy3-dependent autophagy impairment recapitulates presymptomatic neurodegenerative signatures in mice
Source: Sci Rep. 2026 Apr 4;16:16229. doi: 10.1038/s41598-026-43314-0 (PMC13201858; doi:10.1038/s41598-026-43314-0)
Supplement: Supplementary file 1 — Supplementary Material 1 [file 41598_2026_43314_MOESM1_ESM.pdf]

Supplementary Material Guide - *Wdfy3-Dependent Autophagy Impairment Recapitulates Presymptomatic Neurodegenerative Signatures in Mice*

Supplementary Figure Legends

Supplementary Figure 1. Cell and tissue enrichment analysis of mouse cortical proteins

Results of EnrichR analysis against curated gene sets representing various cell types and tissues from 3 databases: Mouse Gene Atlas, PanglaoDB, and Human Gene Atlas. The top 25 enriched cell types and tissues from each database were visualized in a bubble plot using JMP Pro. In this plot, the x-axis shows the negative base-10 logarithm of the adjusted p-value (-log10 p-value), indicating enrichment significance. The y-axis shows the combined score, which combines the p-value and z-score to assess the magnitude and consistency of enrichment. The bubble size corresponds to the odds ratio, reflecting the likelihood of association between the identified proteins and specific cell types or tissues. Detailed results, including data from each database, are provided in **Supplementary Tables 2-4**.

Supplementary Figure 2. ***Comprehensive synaptic localization and functional analysis of mouse cortical proteins using SynGO***

Results of SynGO analysis of cortical proteomes to determine associations with subcellular compartments, structures, and biological functions of identified proteins. The analysis utilized an evidence-based, expert-curated resource for synapse function and gene enrichment studies (dataset version: 20231201). From 2,444 unique proteins identified in the cortical proteome (**Supplementary Table 1**), 766 were mapped to 765 unique SynGO-annotated genes. Among these, 727 genes had Cellular Component annotations (**A**), resulting in 1,349 total annotations, and 522 genes had Biological Process annotations (**B**), totaling 874 annotations. Enrichment analysis revealed that 40 Cellular Component terms and 60 Biological Process terms were significantly enriched at a 1% false discovery rate (FDR), considering terms with at least 3 matching input genes. The "brain expressed" background set was selected for this analysis, comprising 18,035 unique genes, of which 1,591 overlap with SynGO-annotated genes. (**C**) Bar chart displaying the 10 most enriched terms of identified proteins annotated in the SynGO_2024 gene set library based on the -log10 *p*-value. Corresponding *p*-values are indicated next to each term, all of which proved to be highly significant. An asterisk (*) next to a *p*-value signifies that the term also has a significantly adjusted *p*-value (≤ 0.05; **Supplementary Tables 5-6** for further details).

Supplementary Figure 3. ***Clustergram visualization of protein-disease associations in mouse cortical proteome using EnrichR and DisGeNET***

Enrichment analysis was conducted to identify protein-disease associations, visualized using Clustergrammer. All 2,444 proteins identified in the cortical proteome were analyzed using EnrichR, which utilized the DisGeNET database. The resulting clustergram displays the top 30 enriched terms as rows and the top 100 input genes as columns. Each cell indicates whether a gene or protein is associated with a specific term. Rows are ordered by clustering, while columns are sorted based on the sum of *p*-values (**Supplementary Table 7** for a complete list of associated conditions).

Index for Workbook of Supplementary Tables

Sheet #	Sheet Name	Associated Figures and Text	Description
1	Norm. proteome murine	Supp Fig 2-3 Fig 1-2	Normalized proteome of each mouse (Abundance of each of the 2444 unique proteins)
2	Assgn. MGA	Supp Fig 2	Complete list of assigned tissue/cell types based on the gene sets within murine cortical proteome (Using the Mouse Gene Atlas database).
3	Assgn. (PanglaoDB)	Supp Fig 2	Complete list of assigned tissue/cell types based on the gene sets within murine cortical proteome (Using the PanglaoDB database).
4	Assgn. (HGA)	Supp Fig 2	Complete list of assigned tissue/cell types based on the gene sets within murine cortical proteome (Using the Human Gene Atlas database)
5	Enrich. (SynGO_2024)	Supp Fig 3C	Complete list of enriched terms according to murine cortical proteome using SynGO_2024 gene set library.
6	Annot. gene (SynGO_2024)	Supp Fig 3A, B	Complete list of human genes matched to murine cortical proteome with annotations for cellular location and biological function
7	Enrich. (DisGeNet)	Fig 1	Complete list of conditions associated with enrichments from murine cortical proteome.
8	DEP t-test and FC HI-WT	Fig 2	The 414 significant differentially expressed proteins (DEP) in murine cortical proteome (HI vs WT) according to t-test along with their fold changes and log2(FC).
9	DEP (WikiPathways_2024)	Fig 2	Complete list of associated diseases/pathway according to 414 DEP in HI vs WT murine cortical using WikiPathways database
10	DEP assc. (KEGG_2021)	Fig 2	Complete list of associated diseases/pathway according to 414 DEP in HI vs WT murine cortical using KEGG database
11	DEP assc. (Human Pheno	Fig 2	Complete list of associated diseases/pathway according to 414 DEP in HI vs WT murine cortical using the Human Phenotype (Monarch) database
12	DEP t_test PD-CTRL	Fig 3	The 1766 DEP between PD and CTRL human fibroblast groups according to t-test.
13	DEP assc. human	Fig 3	Complete list of diseases/pathways associated with the 1766 DEP between human fibroblasts (PD vs CTRL) using multiple databases

14	DEP Human vs murine	Fig 4A	Shared and exclusive DEP between human fibroblasts (PD vs CTRL) and murine cortex (HI vs WT).
15	Shared DEP assc. (DisGeNET)	Fig 4B	Complete list of diseases/pathways associated with 133 shared DEP between human fibroblasts (PD vs CTRL) and murine cortex (HI vs WT) using the DisGeNET database
16	Neuro assc. shared DEP	Fig 4C	Complete list of neurodegenerative diseases and specific associated proteins from 133 DEP analyzed using DisGeNET database. 19 proteins found contributing to at least two associations.
17	PD-databases	Main Text	Characteristics of the publicly available proteomic datasets from PD human brain CSF and brain tissues. The dataset/species, brain region, accession code, and notes for each database.
18	Database-sets	Main Text	All proteins from the publicly available proteomic datasets from PD human brain CSF and brain tissues under their accession codes, as described above.
19	Intersections	Main Text	Intersections between proteins detected in our murine study and the publicly available proteomic databases in human brain and CSF with PD.
20	Comparison-stats	Main Text	Evaluation of the statistical significance of overlapping proteins between our murine study and publicly available proteomic databases in human brain and CSF with PD. Featuring DEP, number of overlapping proteins, odds ratio and p-value.
21	Database-overlap-DAVID	Main Text	Pathways associated with the overlapping proteins from our murine study and the publicly available proteomic databases in human brain and CSF with PD using DAVID alongside statistical values.
22	Top 15 pathways	Main Text	The top 15 pathways associated with the overlapping proteins from our murine study and the publicly available proteomic databases in human brain and CSF with PD according to the number of overlapping proteins. Using DAVID.

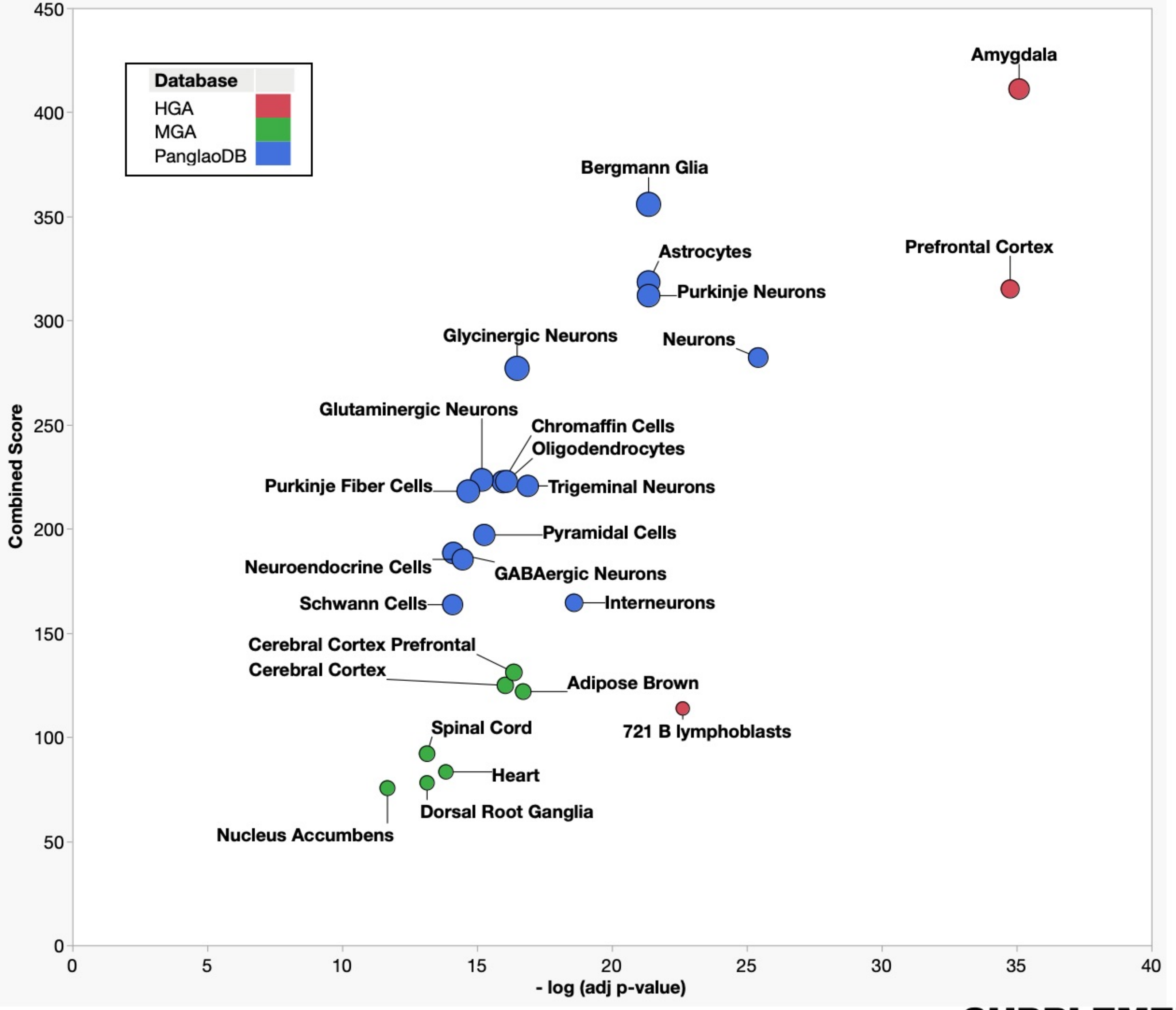

SUPPLEMENTARY FIGURE 1

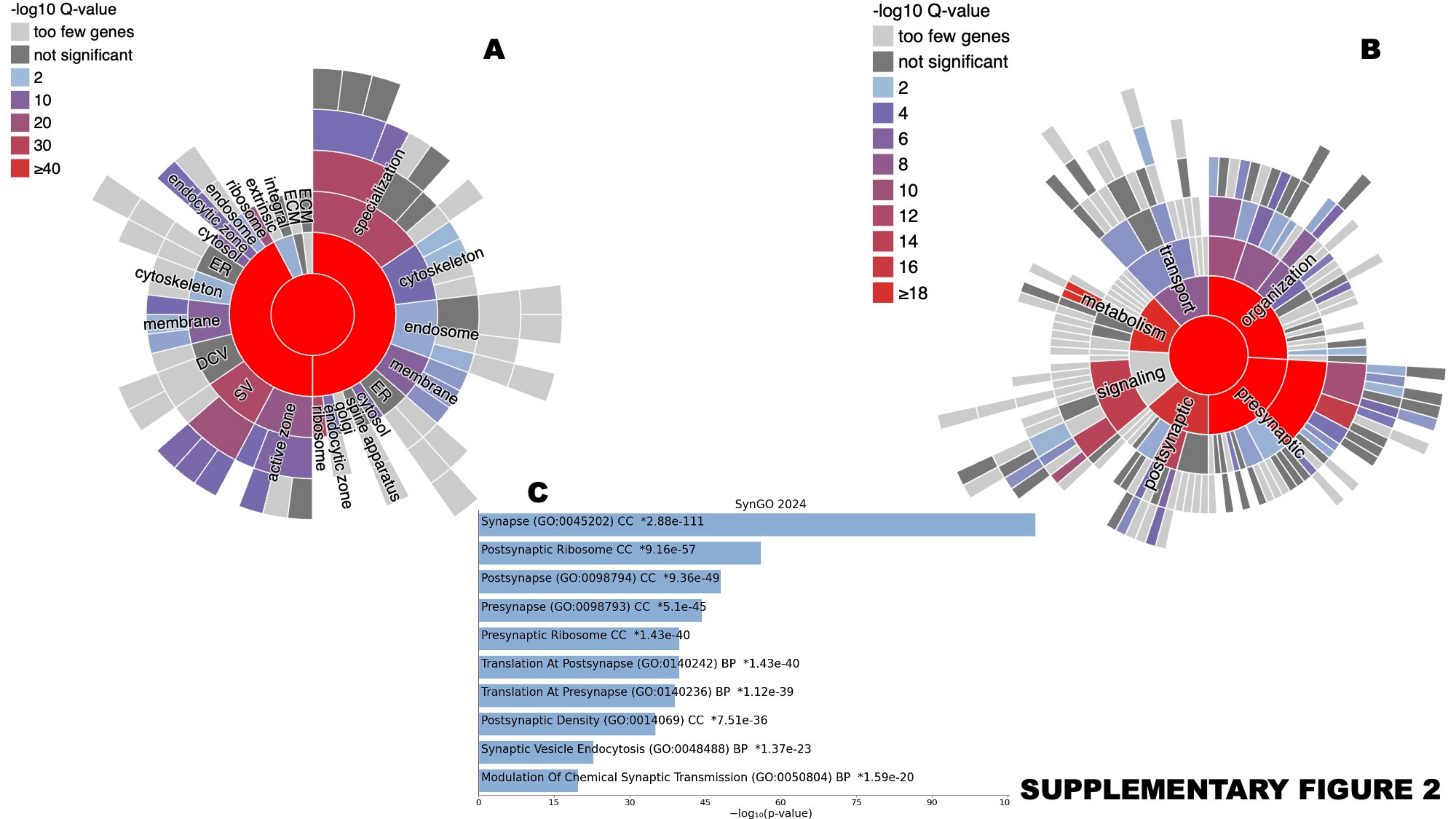

SUPPLEMENTARY FIGURE 2

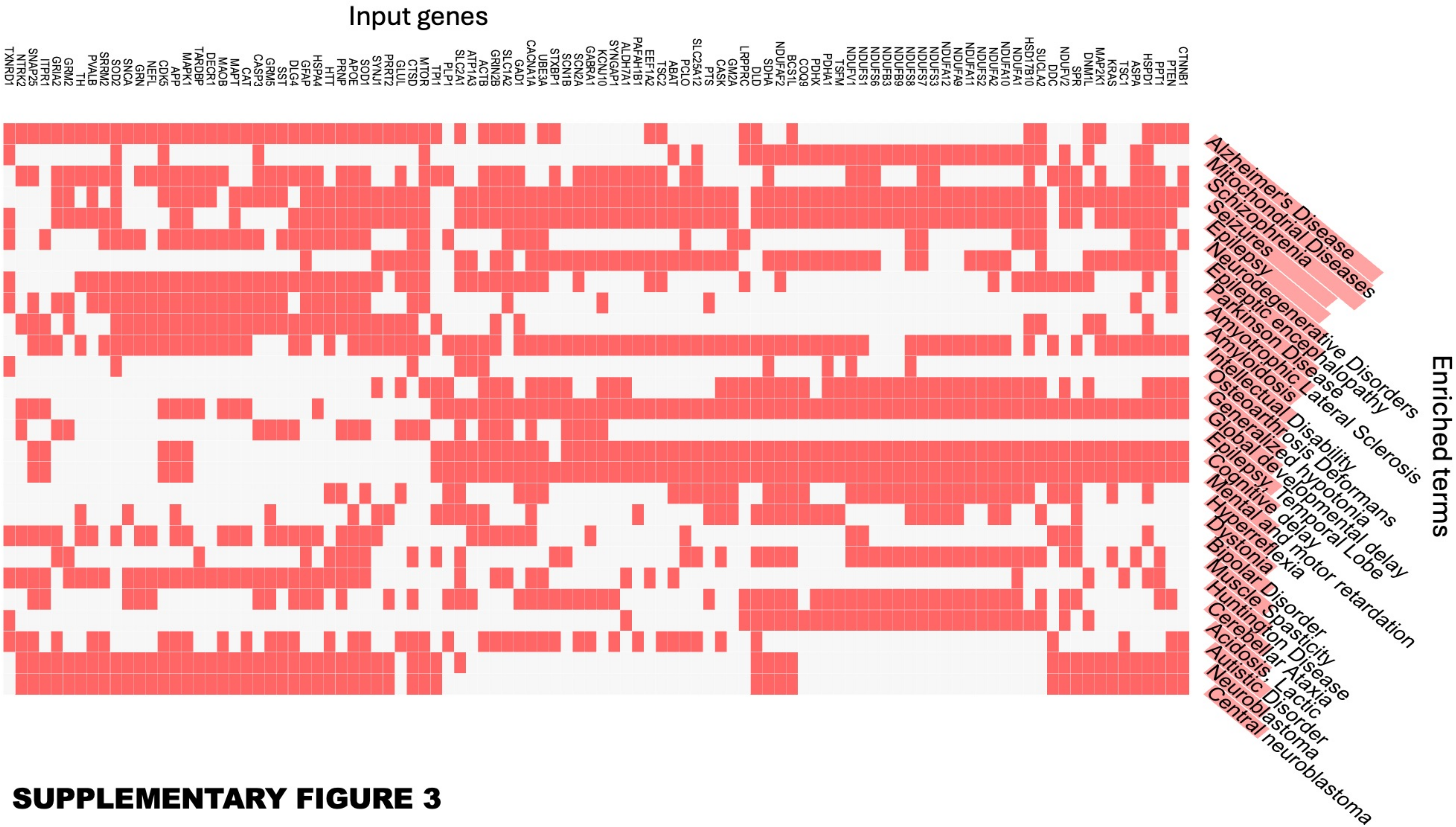

SUPPLEMENTARY FIGURE 3
